# Evaluation of Reference Genes for Transcriptional Profiling in Two Cockroach Models

**DOI:** 10.3390/genes12121880

**Published:** 2021-11-25

**Authors:** Shen Zhu, Yongjun Liu, Mingtao Liao, Yang Yang, Yu Bai, Na Li, Sheng Li, Yunxia Luan, Nan Chen

**Affiliations:** 1 Guangdong Provincial Key Laboratory of Insect Developmental Biology and Applied Technology, Institute of Insect Science and Technology, School of Life Sciences, South China Normal University, Guangzhou 510631, China; 2019022490@m.scnu.edu.cn (S.Z.); 2019022481@m.scnu.edu.cn (Y.L.); 2019022479@m.scnu.edu.cn (M.L.); 2021023047@m.scnu.edu.cn (Y.Y.); yubai@m.scnu.edu.cn (Y.B.); lina5hs@m.scnu.edu.cn (N.L.); lisheng@scnu.edu.cn (S.L.); yxluan@scnu.edu.cn (Y.L.); 2 Guangmeiyuan R&D Center, Guangdong Provincial Key Laboratory of Insect Developmental Biology and Applied Technology, South China Normal University, Meizhou 514000, China

**Keywords:** cockroaches, reference genes, qPCR normalization, gene expression, functional genomics

## Abstract

The German cockroach, *Blattella germanica*, and the American cockroach, *Periplaneta americana* are the most common and synanthropic household pests of interest to public health. While they have increasingly served as model systems in hemimetabolous insects for studying many biological issues, there is still a lack of stable reference gene evaluation for reliable quantitative real-time PCR (qPCR) outputs and functional genomics. Here, we evaluated the expression variation of common insect reference genes, including the historically used *actin*, across various tissues and developmental stages, and also under experimental treatment conditions in these two species by using three individual algorithms (geNorm, BestKeeper, and NormFinder) and a comprehensive program (RefFinder). *RPL32* in *B. germanica* and *EF1*α in *P. americana* showed the overall lowest variation among all examined samples. Based on the stability rankings by RefFinder, the optimal but varied reference genes under specific conditions were selected for qPCR normalization. In addition, the combination of *RPL32* and *EF1*α was recommended for all the tested tissues and stages in *B. germanica*, whereas the combination of multiple reference genes was unfavorable in *P. americana*. This study provides a condition-specific resource of reference gene selection for accurate gene expression profiling and facilitating functional genomics in these two important cockroaches.

## 1. Introduction

The Blattaria cockroaches have evolved as an ancient and highly successful form of insect life. Some species (less than 1%) in this group serve as public health pests, of which the German cockroach, *Blattella germanica*, and the American cockroach, *Periplaneta americana,* are the most common and troublesome household pests worldwide [1]. These two species are strictly synanthropic and usually infest human-built structures, including homes, apartments, restaurants, hospitals, and other places where food is available. They harbor and mechanically transmit various pathogens and trigger asthma and allergic diseases [2,3]. During the last decades, an increasing number of studies on *B. germanica* and *P. americana* have shown them to be valuable organisms for exploring a variety of biological issues. In particular, they serve as model systems for studies of developmental biology and endocrinology in hemimetabolous insects [4,5,6,7,8,9,10] and nutrition and reproduction physiology [11,12,13,14]. As excellent chemical communicators, they have long served as important models for studying chemical ecology, especially in the aspects of sex and aggregation pheromones [15,16,17,18,19,20]. These omnivorous cockroach species are also wildly used for examining host-gut microbiota interactions with regard to their development and behavior [21,22,23,24].

Quantitative real-time polymerase chain reaction (qPCR) is a powerful molecular tool that allows the detection and measurement of messenger RNA (mRNA) at the transcriptional level. Being a faster and more sensitive method over the traditional northern blotting and semi-quantitative PCR, qPCR has developed as the most widely used approach for gene expression profiling and validation of transcriptome data [25,26,27,28]. The accuracy and reliability of qPCR outputs strongly depend on many biological and technical factors, such as sample quality, RNA integrity, cDNA synthesis efficiency, and laboratory procedures involved. Therefore, normalization of the data with appropriate reference genes, also known as housekeeping genes, is needed for minimizing variability [29]. Ideal reference genes are assumed to have constant and stable expression across biotic and abiotic factors. However, it is hard and almost impossible to use universal reference genes under all conditions (e.g., developmental stages, tissues, and experimental treatments). Evaluation and identification of appropriate reference genes prior to qPCR analyses is hence crucial for normalization. Importantly, this is also an indispensable step of the MIQE guideline that currently serves as the golden criteria of qPCR experiments [29].

With the advent of next-generation sequencing technology, many research fields in entomology have been profoundly developed into the Genomic Era. In 2018, the genomes of both *B. germanica* and *P. americana* were published [9,30]. Depending on the genome availability, functional genomic studies could provide an in-depth understanding of cockroaches and novel insights into old issues at the molecular level and on a genome-wide scale. Assessment of gene function by silencing gene expression (e.g., highly efficient RNAi in cockroaches) and accurate measurement of gene expression are needed for successful functional genomics. Hitherto, only *actin* has been used historically and extensively as a reference gene for qPCR normalization in these two species [6,7,8,9,10,11,12,13,14,28,31,32,33]. However, its stability under specific experimental conditions was not empirically validated, yet there is no stable reference gene quantification system for *B. germanica* and *P. americana*.

The goals of this study were to evaluate the stability of candidate reference genes and determine the optima for the accurate quantification of genes of interest across various tissues and developmental stages, as well as under different experimental treatments in *B. germanica* and *P. americana*. We also determined the combination of multiple reference genes in a given tissue and at a specific developmental stage. Our data overall provide condition-specific recommendations as to which reference genes should be selected for expression profiling and functional genomics in these two synanthropic cockroaches.

## 2. Materials and Methods

### 2.1. Cockroach Rearing and Sample Preparation

The lines of *B. germanica* and *P. americana* used in this study have been previously described [9,28]. Both colonies were kept in plastic jars or boxes with in-built egg cartons at ~70% relative humidity under a 12:12 h light/dark photoperiod. *B. germanica* and *P. americana* colonies were separately cultured at 27 °C and 30 °C, respectively. They were provided with commercial rat chow and water ad libitum. For harvesting cockroaches with synchronized development, newly hatched progenies during a 2-day period were transferred into new containers. Freshly emerged nymphs and adults were separated by sex from the colony on the day of molting (day 0) and cultured in groups.

We adopted a sampling strategy involving two nymphal stages and four developmental stages in adulthood, each of which was sampled with various tissues. Note that *P. americana* contains as many as 14 nymphal instars, and the last three instars (N12–14) serve as a key period for metamorphosis, resembling the last nymphal instar (N6) in *B. germanica*. Therefore, mixed specimens of N12–14 (last nymphal instars, LN), as well as the non-metamorphic N9–11 (middle nymphal instars, MN), were sampled for nymphs of *P. americana*. For *B. germanica*, the N5 and N6 stages were selected for the pre-metamorphosis and metamorphosis periods, respectively. For both *B. germanica* and *P. americana* adults, the antenna, head, wing, leg, abdominal integument, gut, and ovary were dissected from female cockroaches on days 1, 3, 5, and 7 (FD1–7, the first vitellogenic cycle), whereas for nymphs the wing and ovary were not sampled. Internal tissues were collected under the protection of RNA*later* Solution (Thermo Fisher Scientific, Vilnius, Lithuania). Four biological replicates were sampled for each tissue at a given developmental stage, and at least five cockroach individuals were used for each sample.

### 2.2. Candidate Reference Genes and Primer Design

Depending on the availability of sequence and genome annotation, six and five commonly used reference genes in other insect species were selected as candidate reference genes for *B. germanica* and *P. americana*, respectively, including the previously reported *actin* gene. Specifically, *glyceraldehyde-3-phosphate dehydrogenase* (*GAPDH*, PSN54931.1), *elongation factor-1-alpha* (*EF1*α, KX228232.1), *actin 5c* (*Actin*, AJ862721.1), *ribosomal protein L32* (*RPL32*, C0J52_12160), *ribosomal protein S23* (*RPS23*, PSN46372.1), and *28S ribosomal RNA* (DQ874201.1) were selected for *B. germanica* (Table S1), and *GAPDH* (JN411914.1), *EF1*α (PaOGS02446), *actin* (AY116670.1), *RPS23* (KJ472479.1), and *18S ribosomal RNA* (*18S*, AF370792.1) were selected for *P. americana* (Table S2). For each candidate gene, three to five pairs of gene-specific primers were designed for amplification of 80–120 bp fragments from the coding region using the Primer-BLAST online tool.

### 2.3. RNA Isolation, cDNA Synthesis, and qPCR

Total RNA was extracted from different tissues with RNAiso Plus reagents (Takara, Dalian, China) according to the supplier’s instructions. The quantity and integrity of RNA samples were assessed by a NanoDrop One spectrophotometer (Thermo Fisher Scientific, Madison, WI, USA) and agarose gel electrophoresis. An aliquot of 2 µg of the RNA extracts was reverse-transcribed to first-strand cDNA using oligo(dT)s and Reverse Transcription M-MLV (RNase H-) (Takara, Dalian, China). All qPCR experiments were conducted in accordance with the MIQE guidelines [29]. For each reference candidate, a specific primer pair with an optimal amplification efficiency was firstly screened by establishing standard curves with a 4-fold diluted cDNA series, which was derived from a whole-body RNA sample. The length and sequence of each PCR fragment were further validated by 1.2% agarose gel electrophoresis and Sanger-based DNA sequencing (Tsingke Biotech., Guangzhou, China). For expression profiling of the reference candidates, qPCR was performed with biological replicates, each with technical triplicates, on a QuantStudio 6 Flex Real-Time PCR System (Life Technologies Holdings Pte Ltd., Singapore). Each reaction was in 20 µL containing 10 µL of Hieff qPCR SYBR Green Master Mix (Yeasen, Shanghai, China), 8 µL of 20-fold diluted cDNA template, and 1 µL of each forward and reverse primer (10 µM). The thermocycling was under the control of a two-step program from 94 °C for 3 min, followed by 40 cycles of 94 °C for 10 s and 56 °C for 30 s.

### 2.4. Juvenile Hormone (JH) Treatment and RNAi Experiment in B. germanica

JH III (Cayman Chemical, Ann Arbor, MI, USA) solution was prepared in acetone with a final concentration of 20 µg/µL [7]. Adult females were briefly anesthetized with carbon dioxide, and 1 µL of the solution was applied to the prosternum with a syringe on day 3. Acetone was used as a negative control for JH III treatment. After 24 h, the antennae were sampled for qPCR analysis, and the expression of *Kr-h1*, a JH primary response gene, was investigated to validate the effect of JH application.

For knockdown of *fruitless* (*fru*), a master gene controlling male courtship in *B. germanica*, a 303 bp fragment was selected as a DNA template for dsRNA synthesis [31]. The fragment was PCR amplified and cloned into a pGEM-T Easy vector (Promega, Madison, WI, USA), followed by validation of the insertion by DNA sequencing. The *Mus musculus lymphotoxin A* gene (*Muslta*) not found in *B. germanica* served as an unrelated control for RNAi [28]. DsRNA synthesis and purification were performed using a T7 RiboMAX Express RNAi System (Promega, Madison, WI, USA) as described in our previous study [28,33]. Cockroaches were anesthetized with carbon dioxide, and 2 µL of dsRNA was injected into the hemocoel at a dose of 6 µg per cockroach. The microinjection of dsRNA was implemented by using a NanoFil syringe (35 G beveled needle) coupled with an ALC-IP600 precision syringe pump (Alcott Biotech., Shanghai, China). Cockroaches received two injections of dsRNA, each on day 0 and day 3, and the head (without antennae) was dissected for qPCR analysis of *fru* expression on day 5.

### 2.5. Inhibitor and Antibiotic Treatment in P. americana

Two inhibitors, including LY294002 and rapamycin (both from MedChemExpress, Monmouth Junction, NJ, USA), were used for inhibiting the activities of phosphoinositide 3-kinase (PI3K) and target of rapamycin (TOR), respectively, via artificial feeding. For both sterile tap water and artificial diet (70% corn flour, 15% wheat bran, 10% bean pulp, and 5% fish meal), either LY294002 or rapamycin was added at a final concentration of 50 or 100 μg/mL(g), respectively. In a separate experiment, a mixture of antibiotics was added into both the water and diet for the establishment of a germ-free strain. The final concentration was 0.25 mg/mL(g) for rifampicin and norfloxacin, and 0.5 mg/mL(g) for gentamicin, doxycycline, ciprofloxacin, streptomycin sulfate, and metronidazole. Newly hatched cockroaches were allowed to feed on the prepared water and food containing either an inhibitor or a mixture of antibiotics for 60 days, during which the water and food were replaced every two days. The heads and guts were dissected for the inhibitor and antibiotic treatments, respectively.

### 2.6. Data Analysis and Statistics

The expression stability of candidate reference genes was evaluated by a panel of different algorithms, including geNorm, BestKeeper, NormFinder, and the comprehensive RefFinder program. GeNorm determines the gene expression stability value, M, by calculating the average pairwise variation of a reference gene to all the other genes included in the same analysis [34]. BestKeeper estimates the stability of a candidate gene based on the standard deviation of C_t_ values and the repeated correlation coefficient of variation (CV) [35]. Despite measuring the overall stability, NormFinder introduces an ANOVA-derived model to calculate both intra- and inter-group variation and ranks reference genes by stability value (SV) [36]. The final composite ranking of stability was determined by a comprehensive web-based analytic tool, RefFinder, which integrates the results obtained by geNorm, BestKeeper, NormFinder, and the comparative delta C_t_ method [37], and then ranks the candidate reference genes based on the geometric mean values (GM) [38]. In all of the mentioned algorithms, a lower value of M, CV, SV, or GM indicates a higher stability or lower variation, and thus, a better reference gene. For recommendation of the optimal number of reference genes required for a robust normalization, geNorm also estimates the pairwise variations (V_n/n+1_, n indicates the number of reference genes) after introducing an additional reference gene, with a cutoff value of M = 0.15. The significant difference in gene expression level between the two groups was determined by Student’s t-test using the IBM SPSS 19.0.

## 3. Results

### 3.1. Validation of Primer Sets

As a first step towards the evaluation of expression stability, we screened and verified appropriate primer pairs by performing melting curve and standard curve analyses. Each of the obtained primer pairs for qPCR amplification yielded a single peak from the melting fluorescence and a single band of the expected size in agarose gel (Figure S1), suggesting the absence of any non-specific product. Meanwhile, a standard curve was established for each primer pair. All the linear correlation coefficients (R^2^) exceeded 0.99, with an amplification efficiency (E%) that varied from 91% to 95% for *B. germanica* and 87% to 98% for *P. americana* (Tables S1 and S2). These data suggest a successful screening of highly specific and efficient primers.

### 3.2. Transcriptional Profiles of Candidate Reference Genes

**Figure 1:**
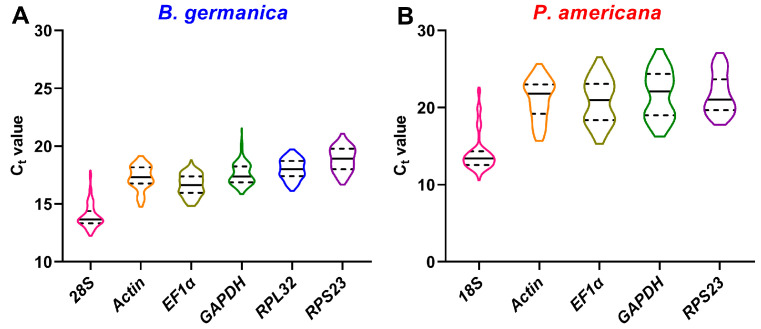
C_t_ value distribution of candidate reference genes in all examined samples from *B. germanica* (**A**) or *P. americana* (**B**). The median and quartiles are indicated by dashed and solid lines.

For *B. germanica,* regarding various different tissues and stages, all calculated C_t_ values of the six candidate genes ranged from 12.3 to 21.5, with *28S* showing the overall highest expression, whereas *RPS23* was the lowest (Figure 1A). Based on the results of four individual algorithms and a comprehensive program, *RPL32* was most stable with the lowest variations among all the samples (Table 1). In the *P. americana* system, the five candidate genes resulted in C_t_ values varying from 10.6 to 27.6. Among them, *18S* showed the highest expression level, and *RPS23* showed the lowest expression (Figure 1B). Evaluation of their stability by geNorm, NormFinder, and RefFinder resulted in *EF1*α being ranked as the most stable reference gene, while *RPS23* showed the lowest variation according to the BestKeeper result (Table 2).

### 3.3. Expression Stability of Candidate Reference Genes throughout Various Stages in Specific Tissues

**Figure 2:**
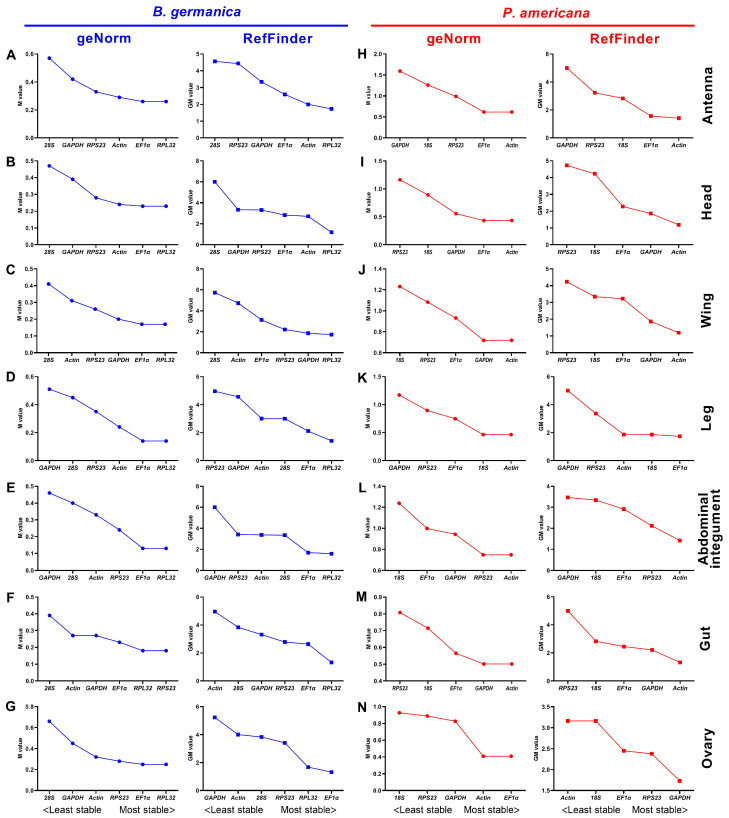
Expression stability rankings of candidate reference genes across different developmental stages in specific tissue types. The M and GM values were calculated by the geNorm and RefFinder algorithms, respectively, for candidate reference genes across different stages in the tissue of antenna, head, wing, abdominal integument, gut, and ovary in either *B. germanica* (**A**–**G**) or *P. americana* (**H**–**N**).

Instead of examining the overall expression variation, we also evaluated the expression stability of these candidate gene expressions under specific conditions. The expression variations throughout various developmental stages were first analyzed in specific tissues by four algorithms. In *B. germanica*, geNorm analysis showed that *RPL32* and *EF1*α were ranked as the most stably expressed genes in most of the examined tissues except for the gut, in which *RPL32* and *RPS23* were the two best choices (Figure 2A–G, left). This is not the case for the BestKeeper algorithm, from which *RPL32* was only recommended in the head, whereas *GAPDH* and *28S* were the best in the other tissues. As for NormFinder, *RPL32* was also preferred in most of the tissues but not the wing, in which *RPS23* was the best (Figure S2A–G). According to the comprehensive ranking by the RefFinder algorithm, *RPL32* was recommended as the most appropriate reference gene in the antennae, head, wing, leg, abdominal integument, and gut of *B. germanica*, whereas *EF1*α was recommended in the ovary (Figure 2A–G, right).

In the case of *P. americana*, geNorm analysis showed that the expression of *actin* throughout different developmental stages was most stable in all the examined tissues, exhibiting a quite similar variation with *EF1*α in the ovary (Figure 2H–N, left). Similarly, either *actin* or *EF1*α was favored by NormFinder in most tissues but not the ovary, in which *GAPDH* was optimal. However, *actin* was preferred by BestKeeper only in the wing and leg, and this algorithm resulted in varied rankings of these genes for the other tissues (Figure S2H–N). A comprehensive ranking by RefFinder showed that *actin* was the optimal reference gene for qPCR normalization in the antennae, head, wing, abdominal integument, and gut of *P. americana*, whereas *EF1*α and *GAPDH* were the best choices for the leg and ovary, respectively (Figure 2H–N, right).

### 3.4. Expression Stability of Candidate Reference Genes across Various Tissues at Given Stages

**Figure 3:**
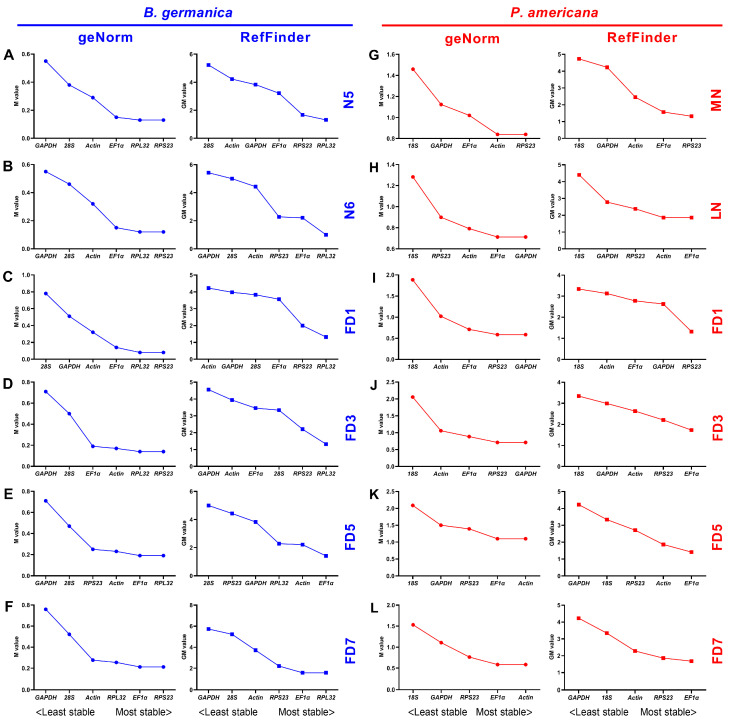
Expression stability rankings of candidate reference genes across various tissues at given developmental stages. The M and GM values were calculated by the geNorm and RefFinder algorithms, respectively, for candidate reference genes across various tissue types at the developmental stages of nymphs N5 (**A**) and N6 (**B**), and adults FD1 (**C**), FD3 (**D**), FD5 (**E**), and FD7 (**F**) in *B. germanica*. In *P. americana*, gene expression stability was calculated at the developmental stages of nymphs MN (**G**) and LN (**H**), and also adults FD1 (**I**), FD3 (**J**), FD5 (**K**), and FD7 (**L**).

We also investigated the expression variations of the candidate reference genes among different tissues at specific developmental stages. In the *B. germanica* system, geNorm analysis resulted in *RPS23* being most stable across various tissues at N5, N6, FD1, FD3, and FD7, while *RPL32* was preferred only at FD5 (Figure 3A–F, left). By contrast, both BestKeeper and NormFinder algorithms obtained varied results, from which either *RPL32*, *RPS23*, *GAPDH*, *EF1*α, or *28S* could be selected at a specific stage (Figure S3A–F). Comprehensive rankings by the RefFinder algorithm selected *RPL32* as the most appropriate reference gene at N5, N6, FD1, FD3, and FD7, while *EF1*α was the best choice at FD5 (Figure 3A–F, right).

As for *P. americana*, geNorm ranked *GAPDH* most frequently at specific stages, with *18S* showing the lowest stability at all stages (Figure 3G–L, left). Based on the BestKeeper algorithm, *RPS23* and *18S* were ranked as the best, while *EF1*α was most frequently favored by NormFinder (Figure S3G–L). According to the summarized ranking of these candidate genes by RefFinder, *EF1*α was the most appropriate at LN, FD3, FD5, and FD7, and *RPS23* was the best at both MN and FD1 stages (Figure 3G–L, right).

### 3.5. Expression Stability of Candidate Reference Genes under Experimental Treatment Conditions

**Figure 4:**
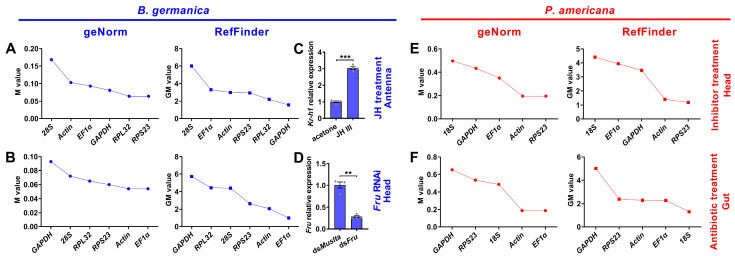
Expression stability rankings of candidate reference genes under experimental treatments. The M and GM values were calculated by the geNorm and RefFinder algorithms, respectively, for candidate reference genes under JH treatment (**A**) or RNAi condition (**B**) in *B. germanica*, and under inhibitor (**E**) or antibiotic (**F**) feeding condition in *P. americana.* Effects of JH and dsRNA treatment in *B. germanica* on the expression of *Kr-h1* (**C**, *n* = 4) and *fru* (**D**, *n* = 4) expression, with normalization to *GAPDH* and *EF1*α, respectively. ** *p* < 0.01, *** *p* < 0.001 (Student’s *t*-test).

To investigate whether the expression of reference genes varies with specific experimental treatments, we next evaluated their stability under specific conditions, including hormone and dsRNA treatments in *B. germanica*, and inhibitor and antibiotic treatments in *P. americana*. For *B. germanica* with JH treatment (Figure 4C), the four algorithms resulted in varied rankings of candidate genes, among which *GAPDH* was favored by the comprehensive RefFinder analysis (Figure 4A and Figure S4A). Under RNAi conditions (Figure 4D), however, all four analyses obtained consistent results showing *EF1*α was the best (Figure 4B and Figure S4B). As for *P. americana*, *RPS23* showed the highest stability among inhibitor treatments according to all four algorithms (Figure 4E and Figure S4C). Differently, *18S* was among the most frequently selected genes under antibiotic treatment (Figure 4F and Figure S4D).

### 3.6. Optimization of Gene Numbers Needed for qPCR Normalization

**Figure 5:**
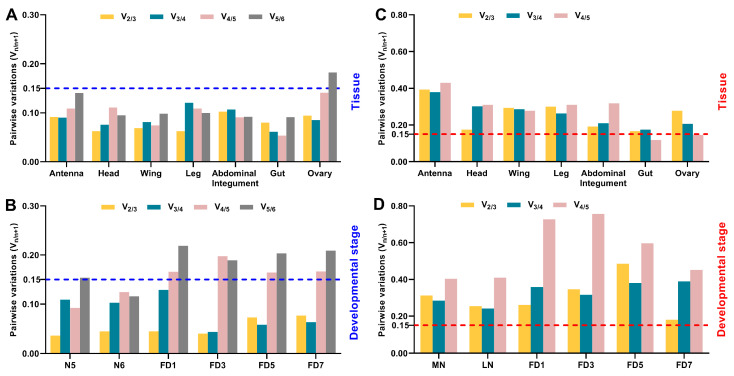
Evaluation of the optimal number of reference genes required for spatiotemporal expression profiling. The pairwise variation V_n/n+1_, where n is the number of reference genes, was calculated by geNorm algorithm using the C_t_ values: (**A**) from different developmental stages in a specific tissue type of *B. germanica*; (**B**) from different tissues at a given developmental stage in *B. gremanica*; (**C**) from different developmental stages in a specific tissue type of *P. americana*; (**D**) from different tissues at a given developmental stage in *P. americana*.

The combination of multiple reference genes is increasingly encouraged to reduce biased normalization, and importantly, this is also required in the MIQE guidelines [29]. To evaluate the optimal number of genes for accurate normalization, we further performed geNorm analyses to calculate the pairwise variations between ranked genes (V_n/n+1_) by successively adding reference genes derived from the RefFinder results in Figure 2 and Figure 3. In all the examined tissues of *B. germanica*, the V_2/3_ values among different stages were lower than 0.15, whereas the inclusion of the least stable gene (*GAPDH*) in the ovary resulted in the V_5/6_ being higher than 0.15 (Figure 5A). These data suggest that the utilization of two reference genes was sufficient for standardizing these samples. Based on the ranking from Figure 2, the combination of *RPL32* and *actin* was recommended for qPCR analysis in the antennae and head, using *RPL32* and *GAPDH* was the optimal combination for the wing, and the combination of *RPL32* and *EF1*α was ideal for the other tissues.

Similarly, at all the examined developmental stages, the V_2/3_ values from different tissues were apparently lower than 0.15. In addition, the inclusion of the fifth reference at most of the stages (e.g., *GAPDH* at FD1) caused significantly higher V_4/5_ values (Figure 5B). Therefore, using two reference genes would standardize these samples well. According to the RefFinder ranking from Figure 3, it is suggested that the combination of *RPL32* and *RPS23* was appropriate for qPCR normalization at N5, FD1, and FD3, using *RPL32* and *EF1*α was the optimal combination at N6 and FD7, and the combination of *EF1*α and *actin* was ideal at FD5. For *P. americana* samples from specific tissues or developmental stages, all V_n/n+1_ values were higher than or very close to 0.15 (Figure 5C,D), suggesting that no appropriate, tested combinations of reference genes can be used in *P. americana*.

Overall, using two reference genes together was efficient for normalizing samples from different tissues and stages in *B. germanica*, and the combination of *RPL32* and *EF1*α was ideal for most tissues and stages. Beyond this case, it is of note that additional inclusion of *EF1*α in the antennae, head, and wing, or at N5, FD1, and FD3, caused insignificant changes in the corresponding variations, nor did the inclusion of *RPL32* at FD5 (Figure 5A,B). Therefore, the combination of *RPL32* and *EF1*α was recommended for all tested spatiotemporal conditions in *B. germanica*.

## 4. Discussion

Many qPCR studies have reached a consensus that it is unrealistic to find a ‘universal’ reference gene showing constant expression across all species and experimental conditions [25]. Identification of appropriate reference genes under different conditions (spatiotemporal and experimental treatments) is therefore mandatory for reliable qPCR analysis in a given species [29]. Hitherto, a stable reference gene system has been established in a variety of insect orders but not the Blattaria cockroaches. In the present study, we evaluated the stability of several reference genes in *B. germanica* and *P. americana*, which are important model systems in hemimetabolous insects. We sampled several tissues and developmental stages that have their own advantages against others on studying biological issues of interest. For example, the antennae should be preferred for exploring chemosensory mechanisms, as with legs for limb regeneration, and the last nymphal instar for metamorphosis. We found that the obtained C_t_ values from either different tissues, stages, or experimental treatments showed a much higher variation in *P. americana* than those in *B. germanica* (Figure 1, Figure 2 and Figure 3). A possible explanation is that the mixed specimens at MN (N9–11) and LN (N12–14) might introduce higher sample variations since *P. americana* harbors a much longer molting cycle at each instar. Previous studies have demonstrated significant impacts of tissue types and developmental stages on reference gene expression, in some cases, even greater than the experimental treatments [39,40,41]. Based on the comprehensive RefFinder ranking that integrates the results of four individual algorithms, *RPL32* showed the lowest variation in most tissues or at most developmental stages in *B. germanica*. By contrast, *actin* was most frequently preferred in *P. americana* tissue types, whereas *EF1*α performed well at most stages (Figure 2 and Figure 3). It is of note that *actin* was most stably expressed across various developmental stages in the *P. americana* gut but was the least stable gene in the ovary (Figure 2M,N). Nevertheless, these varied data highlight the importance of screening condition-specific reference genes in a given species.

While the *actin* gene has been extensively and empirically used in both *B. germanica* and *P. americana*, regardless of tissue types, developmental stages, and experimental treatments, its stability under specific conditions has never been evaluated. Our data showed that *actin* was rarely selected as the most stable reference gene by all four algorithms (only preferred by NormFinder under JH treatment) (Figure S4A) in *B. germanica*. However, it showed the highest stability in several tissue types, but not at various examined stages in *P. americana* (Figure 2 and Figure S2). Therefore, we conclude that *actin* was not appropriate for gene expression analyses, at least in *B. germanica*, for all the examined tissue types and developmental stages, nor under hormone treatment and RNAi conditions.

Overall, the present study has identified appropriate reference genes across different tissue types, developmental stages, and experimental treatments, including hormone application and dsRNA injection in *B. germanica*, and inhibitor and antibiotic feeding in *P. americana*. In *B. germanica*, we recommend *RPL32* as an appropriate internal control for most spatiotemporal conditions, and the combination of *RPL32* and *EF1*α might be ideal for all the tested tissue types and developmental stages. In addition, *GAPDH* and *EF1*α were recommended for the quantification of gene expression under JH treatment and RNAi conditions, respectively. In *P. americana*, *actin* and *EF1*α were appropriate in most tissue types and developmental stages, respectively, while no efficient reference gene combination was sufficient for spatiotemporal normalization. *RPS23* and *18S* was the best choice under inhibitor and antibiotic treatment, respectively. Clearly, more investigations are needed for qPCR analysis under other experimental conditions not tested at this time. This study is the first step toward facilitating functional genomics and an in-depth understanding of cockroaches from aspects of interest at the molecular level.

## Figures and Tables

**Table 1:** Expression variation and stability ranking of the candidate reference genes using geNorm, BestKeeper, NormFinder, and the comprehensive RefFinder algorithm in *B. germanica*.

Rank	Delta C_t_			geNorm		BestKeeper		NormFinder		RefFinder	
	Gene Name	Average C_t_	SD	Gene Name	M	Gene Name	CV	Gene Name	SV	Gene Name	GM
1	*RPL32*	18.00	0.57	*RPL32*	0.24	*RPL32*	4.03	*RPL32*	0.12	*RPL32*	1.00
2	*EF1*α	16.62	0.60	*EF1*α	0.24	*GAPDH*	4.39	*EF1*α	0.22	*EF1*α	2.00
3	*RPS23*	18.87	0.64	*RPS23*	0.31	*RPS23*	4.69	*RPS23*	0.31	*RPS23*	3.57
4	*Actin*	17.28	0.67	*Actin*	0.36	*EF1*α	4.73	*Actin*	0.37	*Actin*	4.23
5	*28S*	13.94	1.06	*28S*	0.61	*Actin*	4.74	*28S*	0.94	*28S*	4.40
6	*GAPDH*	17.58	1.10	*GAPDH*	0.77	*28S*	5.55	*GAPDH*	0.98	*GAPDH*	4.56

**Table 2:** Expression variation and stability ranking of the candidate reference genes using geNorm, BestKeeper, NormFinder, and the comprehensive RefFinder algorithm in *P. americana*.

Rank	Delta C_t_			geNorm		BestKeeper		NormFinder		RefFinder	
	Gene Name	Average C_t_	SD	Gene Name	M	Gene Name	CV	Gene Name	SV	Gene Name	GM
1	*EF1*α	20.79	1.54	*EF1*α	0.98	*RPS23*	10.38	*EF1α*	0.44	*EF1*α	1.41
2	*Actin*	21.10	1.64	*Actin*	0.98	*Actin*	10.58	*Actin*	0.74	*Actin*	1.68
3	*RPS23*	21.76	1.77	*RPS23*	1.25	*18S*	11.74	*RPS23*	1.10	*RPS23*	3.00
4	*GAPDH*	21.64	1.84	*GAPDH*	1.34	*EF1*α	12.00	*GAPDH*	1.32	*18S*	3.34
5	*18S*	14.07	2.75	*18S*	1.91	*GAPDH*	12.62	*18S*	2.59	*GAPDH*	4.23

## Data Availability

All data are available in the main text and Supplementary Materials.

## Supplementary Materials

The following are available online at https://www.mdpi.com/article/10.3390/genes12121880/s1, Figure S1. Evaluation of the specificity and amplification efficiency of the primer pairs; Figure S2. Expression stability rankings of candidate reference genes across different developmental stages in specific tissue types; Figure S3. Expression stability rankings of candidate reference genes across various tissues at given developmental stages; Figure S4. Expression stability rankings of candidate reference genes under experimental treatments; Table S1. Primers used for evaluation of candidate reference genes in *B. germanica*; Table S2. Primers used for evaluation of candidate reference genes in *P. americana*.
