# Supplementary material for: Evaluation of Reference Genes for Transcriptional Profiling in Two Cockroach Models
Source: Genes (Basel). 2021 Nov 25;12(12):1880. doi: 10.3390/genes12121880 (PMC8701133; doi:10.3390/genes12121880)
Supplement: Supplementary file 1 [file genes-12-01880-s001.zip › genes-1460569-suppl.pdf]

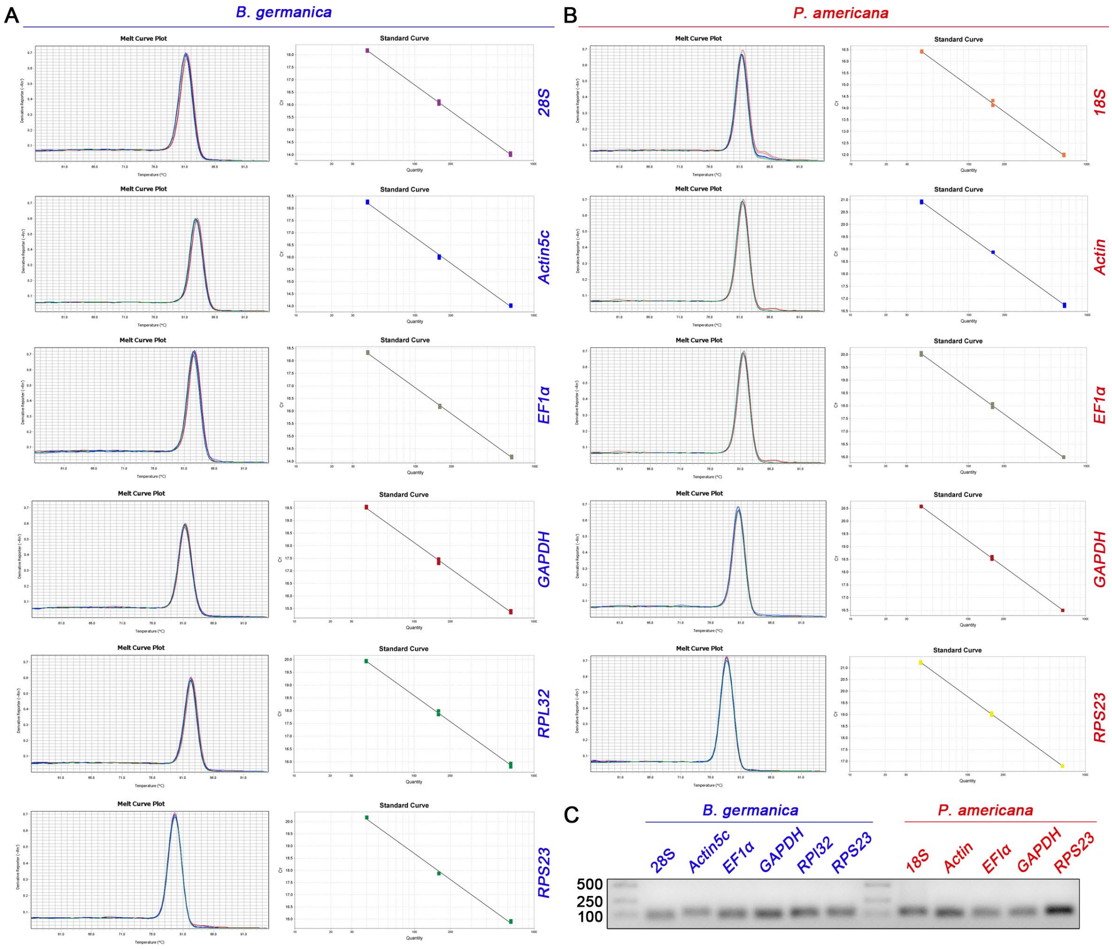

Figure S1. Evaluation of the specificity and amplification efficiency of the primer pairs. The specificity and efficiency of primer sets was determined by melting curve and standard analysis, respectively, and the length of qPCR products was visualized by agarose gel electrophoresis.

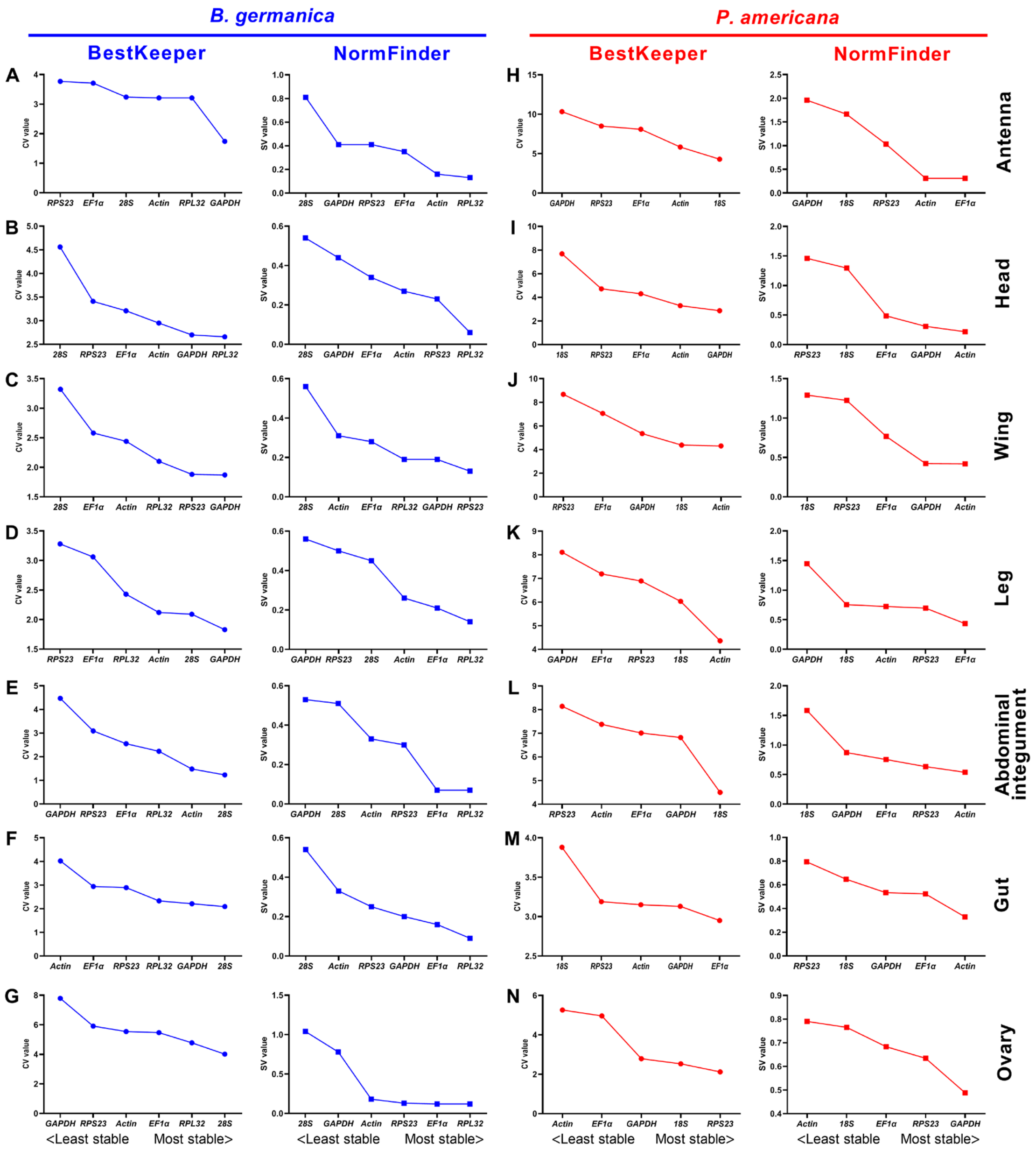

Figure S2. Expression stability rankings of candidate reference genes across different developmental stages in specific tissue types. The CV and SV values were calculated by BestKeeper and NormFinder algorithm, respectively, for candidate reference genes across different stages in the tissue of antenna, head, wing, abdominal integument, gut, and ovary in either *B. germanica* (**A–G**) or *P. americana* (**H–N**).

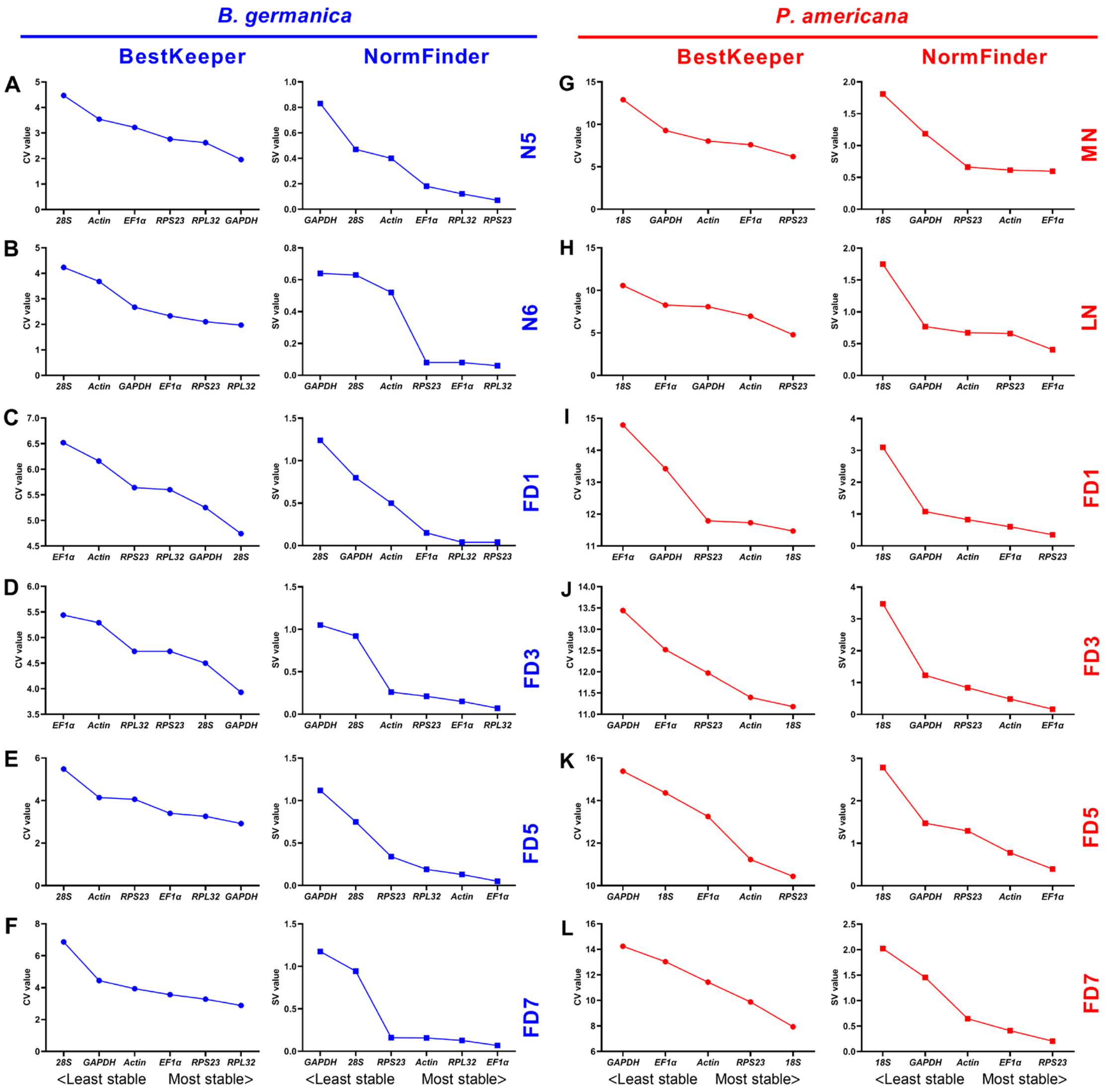

Figure S3. Expression stability rankings of candidate reference genes across various tissues at given developmental stages. The CV and SV values were calculated by BestKeeper and NormFinder algorithm, respectively, for candidate reference genes across various tissue types at the developmental stages of nymphs N5 (**A**) and N6 (**B**), and adults FD1 (**C**), FD3 (**D**), FD5 (**E**), and FD7 (**F**) in *B. germanica*. In *P. americana*, gene expression stability was calculated at the developmental stages of nymphs MN (**G**) and LN (**H**), and also adults FD1 (**I**), FD3 (**J**), FD5 (**K**), and FD7 (**L**).

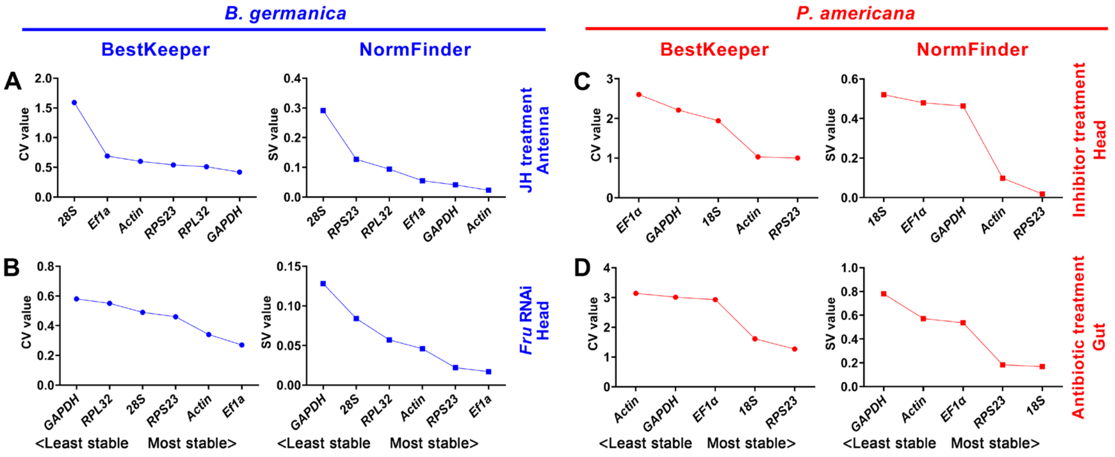

Figure S4. Expression stability rankings of candidate reference genes under experimental treatments. The CV and SV values were calculated by BestKeeper and NormFinder algorithm, respectively, for candidate reference genes under JH treatment (**A**) or RNAi condition (**B**) in *B. germanica*, and under inhibitor (**C**) or antibiotic (**D**) feeding condition in *P. americana*.

Table S1. Primers used for evaluation of candidate reference genes in *B. germanica*.

Gene name	Primer sequences (forward and reverse)	Length of product (bp)	Amplification efficiency (%)	R^2 of standard curve
GAPDH	**F:** CTGTTCCCAATGTGTCTGTT<br>**R:** CTGCCTTTACCTTTGCCTTA	87	95.49	0.998
EF1α	**F:** TGGAACACTCCGACAAGAT<br>**R:** TCAATGAGACACTTGCCTTC	85	95.38	0.999
Actin5c	**F:** TGAGACCACATACAACTCCA<br>**R:** CAATTCCAGGGTACATGGTG	104	92.37	0.998
RPL32	**F:** CAATCATGGAGAAAGCCCAA<br>**R:** TTCCTGTTGCTTCCATAACC	98	97.82	0.999
RPS23	**F:** AAACAGCCAAACAGTGCTAT<br>**R:** CAATGTAGTTCAGGCAACCA	106	91.43	0.997
28S	**F:** ATCGTCAGAGCTGGGTATAG<br>**R:** TATCCTGAGGGAAACTTCGG	80	94.82	0.999

Table S2. Primers used for evaluation of candidate reference genes in *P. americana*.

Gene name	Primer sequences (forward and reverse)	Length of product (bp)	Amplification Efficiency (%)	R^2 of standard curve
GAPDH	**F:** GGCCAGAATATCATACCAGC<br>**R:** CGGAATGCCATACCAGTTAG	95	97.56	0.999
EF1α	**F:** AAGGAAGGAAAAGCTGATGG<br>**R:** AGACGAAGAGGTTTCTCAGT	89	98.35	0.999
Actin	**F:** GCGATTTGACAGACTACCTC<br>**R:** CTCCTTAATGTCACGCACAA	95	94.08	0.999
RPS23	**F:** TTCGGAAGTGTGTGAGAGTA<br>**R:** CCACCAGGACTTCATCATTC	111	86.97	1.0
18S	**F:** AATGGAATAGGACCTCGGTT<br>**R:** AATTTCACCTCTAACGTCGC	87	86.98	0.998
